# COVID-19 vaccination hesitancy in Kazakhstan: Gaukhar Mergenova

**DOI:** 10.1093/eurpub/ckac129.662

**Published:** 2022-10-25

**Authors:** G Mergenova, S Rosenthal, B Zhussupov, A Izekenova, L Alekesheva, A Izekenova, B Iskakova, A Myrkassymova, A Bukharbayeva, A Davis

**Affiliations:** 1 Global Health Research Center of Central Asia, Almaty, Kazakhstan; 2 Kazakh National Medical University, Almaty, Kazakhstan; 3 Department of Pediatrics and Psychiatry, Columbia University Irving Medical Center, New York, USA; 4 University of International Business, New York, USA; 5 Columbia University School of Social Work, New York, USA

## Abstract

**Background:**

COVID-19 vaccine hesitancy is a major problem worldwide that impedes vaccine uptake. We explored factors associated with vaccine hesitancy in Kazakhstan.

**Methods:**

We conducted a cross-sectional face-to-face survey of 991 adults in Kazakhstan in July 2021, using quota sampling of respondents over 18 years old reflecting the distribution of gender, age, residence type, and geographical regions of Kazakhstan, according to the 2020 census.

**Results:**

Over two third (68.4%) of the sample was vaccine hesitant; 22.11% - received a vaccine (18.6%-Sputnik V, 2%-Hayat-Vax, 0.9%-QazCovid and 0.6%- CoronaVac). We used logistic regression to explore factors that were associated with vaccine hesitancy, adjusting age, education, employment, type of residence, self-reported COVID-19. The odds of not being vaccine hesitant were higher among those who had a higher perception that the COVID-19 vaccine was important for health OR = 2.66 (95%CI:2.24,3.17), higher belief in vaccine safety/effectiveness OR = 3.16 (95%CI:2.57,3.89), higher trust in government/health providers OR = 3.32 (95%CI:2.72,4.05), higher trust in official sources of information OR = 1.16 (95%CI:1.12,1.21), higher adherence to preventive measures OR = 1.05 (95%CI:1.03,1.08), knew someone diagnosed with COVID-19 OR = 1.36 (95%CI:1.01,1.82), or who died of COVID-19 OR = 1.47 (95%CI:1.04,2.08), had been ever tested for COVID-19 OR = 1.75 (95%CI:1.30,2.35), had ever received flu vaccine OR = 2.16 (95%CI:1.62,2.88), among health professionals OR = 2.76 (95%CI:1.38,5.51), and who had lower vaccine conspiracy beliefs OR = 0.48 (95% CI:0.40,0.58).

**Conclusions:**

Vaccine accepting individuals held positive beliefs about the COVID-19 vaccine, had greater trust in government/official sources of information/health care workers, had greater exposure to COVID-19. Interventions aimed at reducing vaccine hesitancy need to address sources people find credible and may need to target individuals who have had limited exposure to the risks of COVID-19.

**Key messages:**

• Strategies to increase trust in government/ health care workers and official sources of information can be an effective approach to reduce COVID-19 vaccination hesitancy.

• Interventions to reduce COVID-19 vaccination hesitancy should target individuals who have had limited exposure to the risks of COVID-19.

